# Anterior femoral cutaneous nerve injury following femoral catheterization: A case series and review

**DOI:** 10.1016/j.inpm.2026.100834

**Published:** 2026-11-03

**Authors:** Nimish Mittal, Seungjae Cho, Salmah Hassan, Jordan Farag, Vincenzo Ricci, Kamal Mezian, Ondřej Naňka

**Affiliations:** a Toronto Rehabilitation Institute, University Health Network, Toronto, ON, Canada; b Division of Physical Medicine and Rehabilitation, University of Toronto, Toronto, ON, Canada; c University of Toronto Faculty of Medicine, Toronto, ON, Canada; d International Islamic University Malaysia, Kulliyyah of Medicine, Kuala Lumpur, Selangor, Malaysia; e Luigi Sacco University Hospital, Physical and Rehabilitation Medicine Unit, ASST Fatebenefratelli-Sacco, Milan, Italy; f Charles University First Faculty of Medicine, Department of Rehabilitation Medicine, Prague, Prague, Czech Republic; g General University Hospital in Prague, Prague, Prague, Czech Republic; h Charles University First Faculty of Medicine, Institute of Anatomy, Prague, Prague, Czech Republic

**Keywords:** Anterior femoral cutaneous nerve, Intermediate femoral cutaneous nerve, Femoral catheterization, Nerve hydrodissection, Ultrasound, Neuropathic pain

## Abstract

Femoral vessel catheterization is widely performed for diagnostic and therapeutic indications but carries a small risk of injury to adjacent nerves. Anterior femoral cutaneous nerve (AFCN) injury is an under-recognized cause of persistent anteromedial thigh neuropathic pain after femoral arterial access. We review the anatomy of the AFCN and its branches, the intermediate femoral cutaneous nerve (IFCN) and medial femoral cutaneous nerev (MFCN), together with the mechanisms of injury, clinical presentation, and management, and present two cases of catheterization-related neuropathic pain consistent with intermediate femoral cutaneous nerve (IFCN) involvement that were successfully treated with ultrasound-guided hydro-dissection. Both patients achieved sustained symptom resolution at six months. These cases highlight AFCN injury as a treatable complication of femoral access and support a role for targeted ultrasound-guided hydro-dissection in selected patients.

## 1 Introduction

Femoral vessel catheterization is routinely performed for coronary angiography, vascular intervention, and hemodynamic monitoring, but carries a small risk of injury to adjacent nerves [1]. Anterior femoral cutaneous nerve (AFCN) injury is one such iatrogenic complication. The AFCN is a sensory branch of the femoral nerve that supplies the skin of the anterior thigh to the knee; its superficial course and proximity to the femoral artery in the proximal thigh render it vulnerable during catheter insertion, femoral artery repair, or varicose vein surgery, with scarring or direct injury producing nerve entrapment. Analyzing nearly 16 million transfemoral catheterizations in the United States National Inpatient Sample (2002–2010), El-Ghanem et al. reported femoral neuralgia involving sensory and motor branches in 3.8 per 100,000 patients [2].

## 2 AFCN anatomy

The AFCN comprise the intermediate and medial femoral cutaneous nerves (IFCN and MFCN), which arise from the anterior (superficial sensory) division of the femoral nerve within the femoral triangle, distal to the inguinal ligament (Fig. 1A and Fig. 1B). The IFCN pierces the fascia lata on the sartorius' anterior surface, within the iliopectineal fossa about 7–8 cm distally, then supplies the anteromedial proximal two-thirds of the thigh. The MFCN lies deeper in the iliopectineal fossa, lateral to the femoral artery, then crosses anterior to reach its medial side, dividing near the femoral triangle apex into anterior (MFCN-A) and posterior (MFCN-P) branches. The anterior branch pierces the fascia lata anterior to the sartorius at the upper third of the thigh, distal to the IFCN perforation sites, supplying the anteromedial knee—particularly the medial parapatellar area and total knee arthroplasty incision zones—with some fibers reaching genicular tissues and the medial retinaculum. The posterior branch runs deeper along the medial sartorius near the femoral vessels, saphenous nerve, and nerve to vastus medialis, supplying the posteromedial anteromedial knee skin.

**Fig. 1A:**
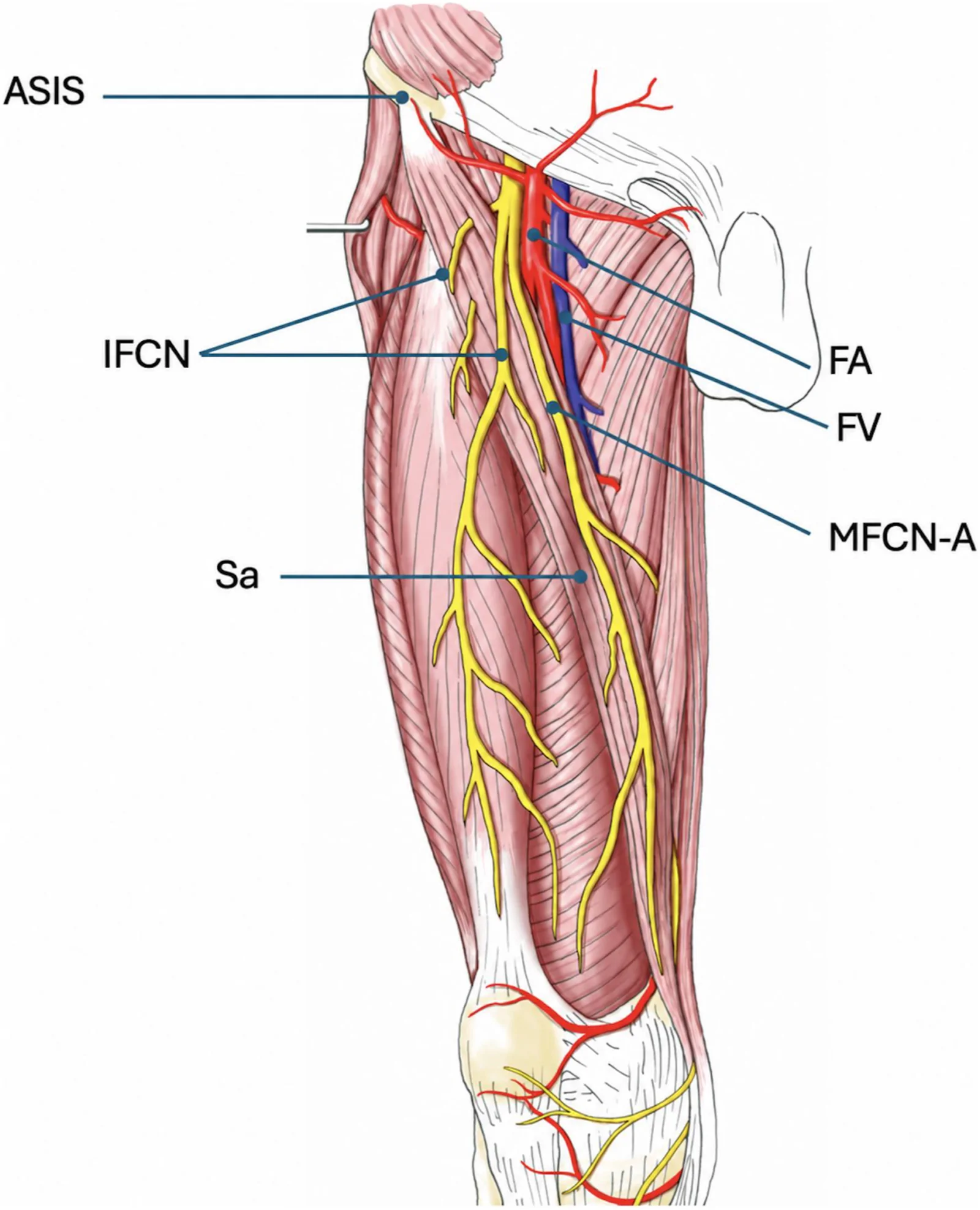
Anatomical illustration of the course of anterior femoral cutaneous nerve branches. ASIS- Anterior superior iliac spine; IFCN - intermediate femoral cutaneous nerve; MFCN-A, anterior branch of the medial femoral cutaneous nerve; FA - Femoral artery; FN - Femoral nerve; Sa - Sartorius muscle.

**Fig. 1B:**
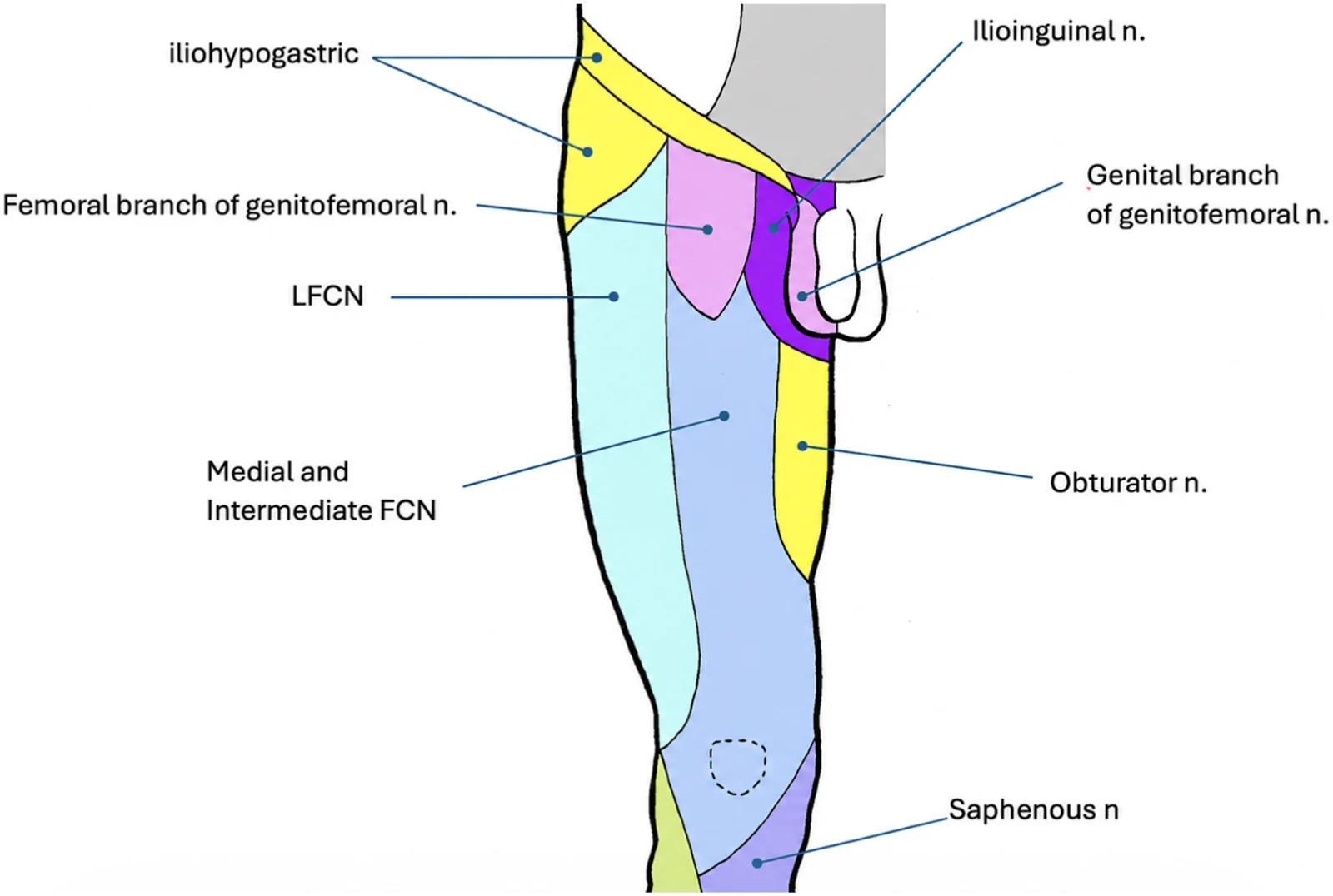
Sensory cutaneous innervation of thigh/leg. LFCN - lateral femoral cutaneous nerve; FCN –femoral cutaneous nerve; n-nerve.

### 2.1 Case reports

***Ethics and Consent***: This report describes a case series of fewer than three patients. All patient information has been fully anonymized; no names, identifiers, institutional dates, or other protected health information are included. All illustrations comprise original drawings, cadaveric dissection images, and de-identified sonoanatomical images from the authors' collection, and contain no patient-identifying information. In accordance with University Health Network Research Ethics Board (REB) guidance, case reports involving fewer than three patients are not eligible for REB review submission and therefore do not require formal REB approval and written informed consent was not required. The manuscript has been prepared in compliance with institutional privacy and confidentiality requirements, and all identifying patient information has been removed.

**Case 1.** A 57-year-old man who had undergone femoral catheterization for cardiac valvuloplasty six months earlier presented with persistent numbness and burning over the anteromedial right thigh, without lower-limb weakness. The pain became chronic, significantly affected his quality of life, and responded poorly to pharmacological treatment, including gabapentin (titrated to 2400 mg/day), pregabalin (200 mg twice daily), and over-the-counter acetaminophen and ibuprofen. Sensory examination showed a 60–80% reduction in light-touch and pinprick sensation over the anteromedial right thigh compared with the left. Motor power was 5/5 (MRC) at hip flexion, knee extension, and ankle dorsi- and plantarflexion; deep tendon reflexes were normal and symmetrical; and the dermatomal and myotomal examination was otherwise unremarkable. Ultrasound-guided diagnostic and therapeutic right IFCN hydro-dissection was performed as a combined diagnostic and therapeutic procedure; the local anaesthetic produced immediate relief of the target pain, confirming the IFCN as the pain generator. Pain decreased from 5/10 before the procedure to 0–1/10 at three months and was maintained at six months, after which he was discharged.

**Case 2.** A 53-year-old man underwent emergent endovascular thrombectomy and carotid stenting for left internal carotid artery dissection with middle cerebral artery infarction, via right femoral access, complicated by a right groin hematoma. He recovered near-complete motor and sensory function but developed central post-stroke pain. Over the following five years his diffuse hemibody pain improved with duloxetine and a buprenorphine patch (average 5–7/10, worst 9/10). This diffuse central pain was mild, constant and non-focal and was not reproducible by local palpation. Separately, a focal neuropathic pain persisted in the right groin and anteromedial thigh with intermittent radiation to the knee; it was sharp and burning, mechanically provoked by light touch (clothes), and confined to the IFCN territory. A right inguinal hernia repaired with mesh gave no relief. On presentation, examination showed near-complete sensory recovery with only residual right hemisensory change, together with focal, mechanically provoked anteromedial thigh pain that was reproducible on palpation at the mid-inguinal ligament and confined to the IFCN territory—distinct from his diffuse central pain—suggesting IFCN involvement. Ultrasound-guided hydro-dissection of the right IFCN and MFCN-A, used as a combined diagnostic and therapeutic procedure, produced immediate relief and complete resolution of the focal thigh pain (0/10) at one month, sustained at six months, while his central pain continued to be managed pharmacologically; he was subsequently discharged from the pain service.

## 3 Clinical presentation and workup

The sensory distribution of the IFCN and MFCN corresponds closely to the neuropathic pain pattern, which aids localization. Patients typically report burning, tingling, or dysesthetic pain in an IFCN or MFCN territory, often following trauma, surgery, or prolonged pressure near the inguinal or femoral region (Fig. 1C). Injury to the femoral branch of the genitofemoral nerve, which runs lateral to the femoral artery and innervates the upper femoral triangle, should also be considered; careful mapping of the distinct sensory territories (Fig. 1C) helps differentiate it from AFCN branch injury. Electrophysiological studies are of limited value given the small sensory caliber of these nerves, so diagnosis rests on clinical assessment, sensory mapping, and a confirmatory response to a diagnostic local anaesthetic injection.

**Fig. 1C:**
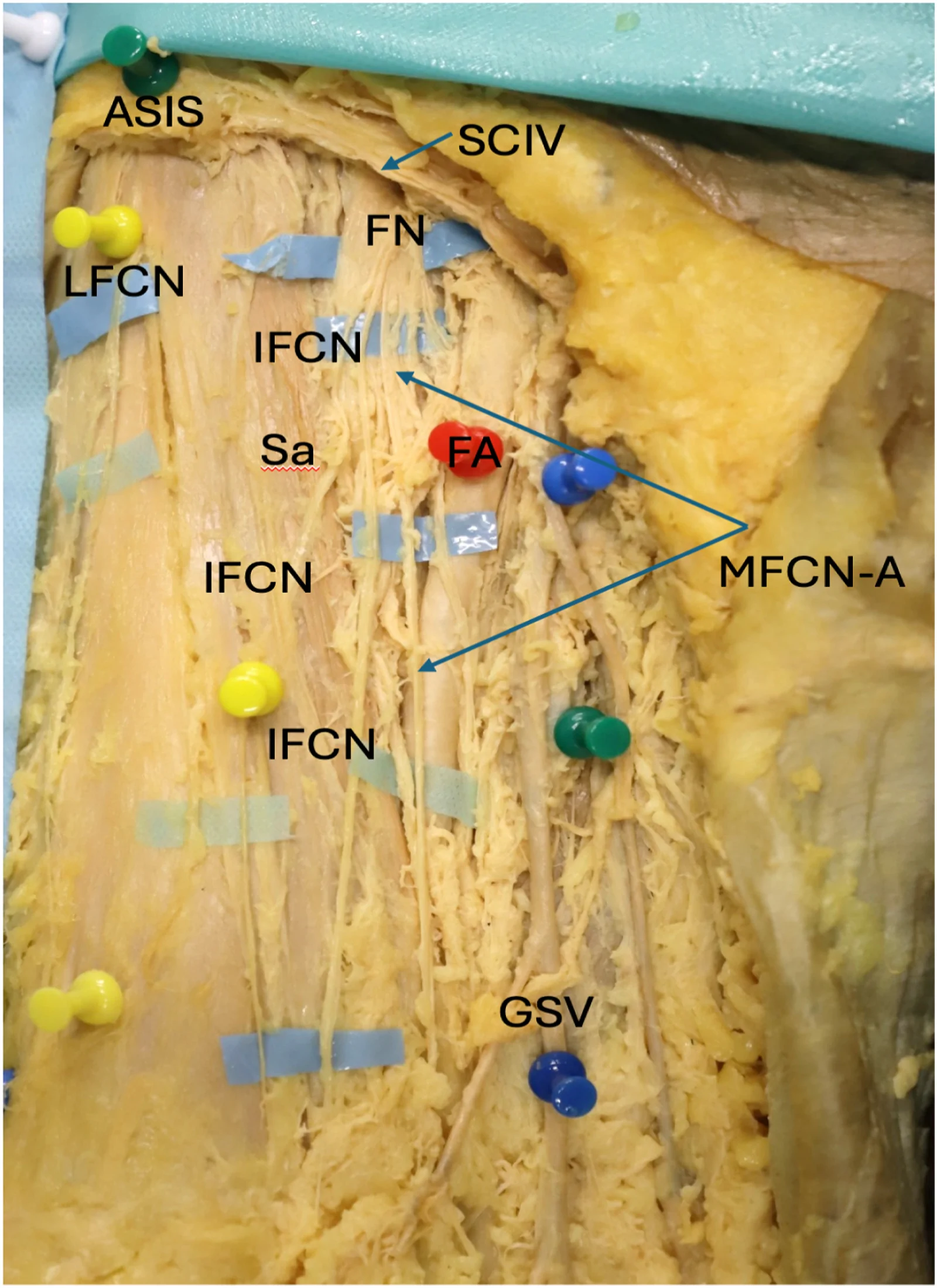
Course of anterior femoral cutaneous nerve and surrounding landmarks in a cadaver. ASIS –anterior superior iliac spine; LFCN – lateral femoral cutaneous nerve; SCIV - Superficial circumflex iliac vein; FN – femoral nerve; IFCN – intermediate femoral cutaneous nerve; Sa- Sartorius muscle; FA – femoral artery; MFCN-A, anterior branch of the medial femoral cutaneous nerve; GSV – Great saphenous vein.

## 4 Ultrasound-guided hydro dissection and nerve block of the IFCN and MFCN

In both cases above, the IFCN was targeted. The sensory distribution of each nerve, together with practical pearls for ultrasound-guided scanning and injection, is summarized in Table 1. In both patients, the perineural injection of steroid and local anaesthetic served as combined diagnostic and therapeutic procedure; the terms perineural block and hydro-dissection are used interchangeably here, with the local anaesthetic providing immediate diagnostic confirmation while the steroid and injectate volume of the same injection delivered therapeutic hydro-dissection. Imaging used a Sonosite LX system (FUJIFILM Sonosite, Bothell, WA, USA) with a 15–4 MHz linear transducer, following the technique of Bjørn et al. [3]. With the patient supine and knees extended, the transducer was placed transversely over the anterior thigh at the level of the anterior superior iliac spine and moved distally to identify the sartorius, with the iliacus visualized deep and medial. Scanning continued in a slightly oblique plane parallel to the inguinal crease until the femoral neurovascular bundle was identified, then toward the apex of the iliopectineal triangle to approximately 5–8 cm distal to the crease. At this level the IFCN appears as a small hyperechoic rounded structure within a characteristic “fat-filled tunnel,” emerging from the medial side of the femoral nerve and piercing the fascia lata anterior to the sartorius; this was the injection target (Fig. 2A).

**Table 1:** Sensory innervation, sonographic localization, injection targets, and procedural tips/cautions for branches of the anterior femoral cutaneous nerve.

Nerve branch	Sensory innervation	Typical ultrasound target/injection site	Key tips/caution
IFCN	Anteromedial proximal to mid-thigh, typically the proximal two-thirds of the anterior thigh	At the point where the nerve appears as a small hyperechoic structure within a “fat-filled tunnel”, near the apex of iliopectineal triangle, as it pierces the fascia lata anterior to the sartorius, usually ∼5–8 cm distal to the inguinal crease	Close to femoral access region; may be vulnerable to iatrogenic injury after femoral cannulation/catheterization
MFCN-A	Distal anteromedial thigh and medial parapatellar/anterior knee region	At or near its fascial duplicature/perforation point anterior or medial to the sartorius, where FA dives down, typically a few centimeters distal to the IFCN target near the apex of the femoral triangle	Variable branching pattern and overlap with adjacent sensory nerves may make localization challenging
MFCN-P	Posteromedial portion of the anteromedial knee region	Usually not targeted; identified in a deeper plane along the medial border of the sartorius near the apex of the femoral triangle, where it may partially encircle the femoral artery	Lies close to the femoral artery, saphenous nerve, and nerve to vastus medialis; avoid routine injection because of risk to adjacent neurovascular/motor structures

**Fig. 2A:**
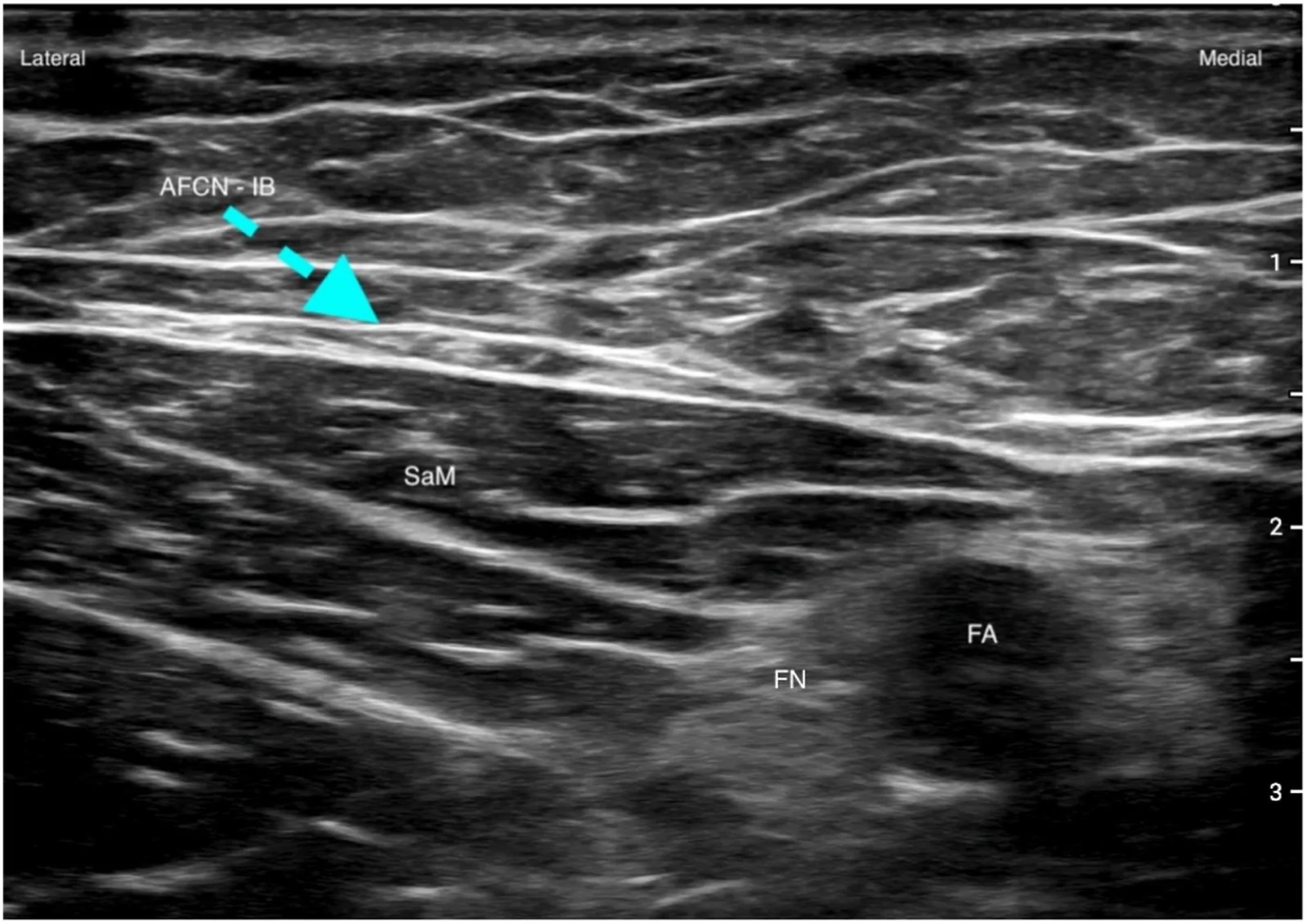
Ultrasound image of injection target for anterior femoral cutaneous nerve intermediate branch. AFCN – IB, Anterior femoral cutaneous nerve intermediate branch; SaM – Sartorius muscle; FN – Femoral nerve; FA – Femoral artery.

For the MFCN, the transducer should be advanced a further 3–5 cm to the level at which the nerve bifurcates near the femoral triangle apex. The anterior branch is visualized within a fascial duplicature of the fascia lata or at its perforation anterior or medial to the sartorius (Fig. 2B). The posterior branch, lying deeper near the femoral artery, is typically not targeted, to avoid unintended motor blockade. After skin preparation with 2% chlorhexidine gluconate, draping, and covering the probe with sterile film, a 25-gauge, 2-inch needle was advanced in-plane from lateral to medial into the perineural plane. Following infiltration with 2 mL of 1% lidocaine, hydro-dissection was performed with a total injectate of 5 mL (40 mg methylprednisolone acetate plus 4 mL of 0.25% bupivacaine), delivered incrementally under continuous ultrasound visualization to separate the nerve from adjacent fascia at both deep and superficial interfaces. Circumferential anechoic fluid surrounding the nerve confirmed adequate release.

**Fig. 2B:**
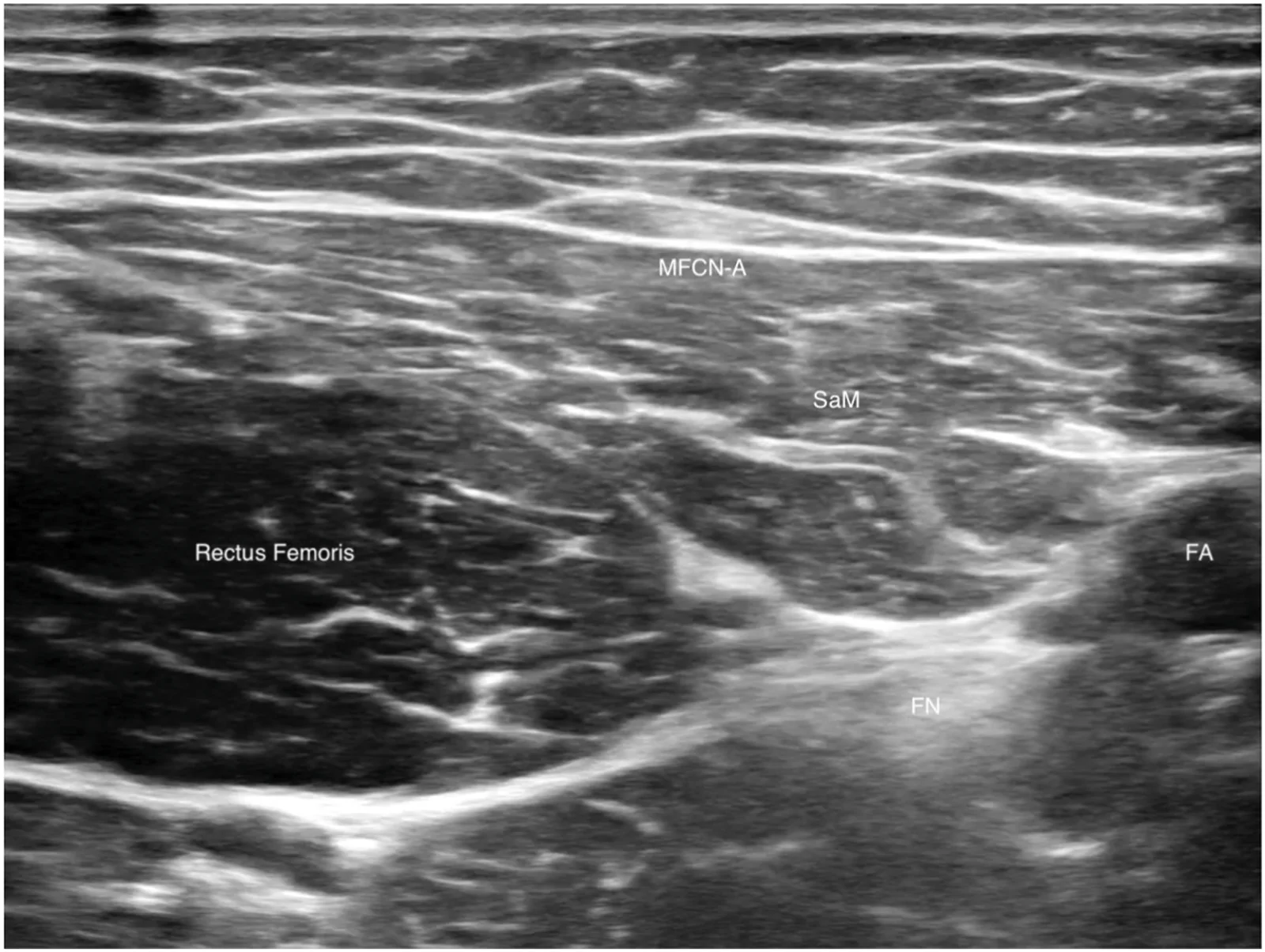
Ultrasound image of injection target for medial femoral cutaneous nerve - Anterior branch. MFCN-A, anterior branch of the medial femoral cutaneous nerve; SaM - Sartorius muscle; FA - Femoral artery; FN - Femoral nerve.

## 5 Discussion

AFCN injury typically results from direct trauma during femoral catheterization, prolonged digital compression to secure hemostasis, or injury to the femoral nerve requiring surgical repair. Larger catheters, multiple puncture attempts, and improper needle angle increase the risk [2], as do pre-existing anatomical variations. External compression by vascular complications, notably femoral pseudoaneurysms and retroperitoneal or groin hematomas, is an important cause [4]. Presentation ranges from mild sensory loss to debilitating pain. Kent et al. reported an average 37-h delay to recognition of neural injury among 9585 catheterizations, with symptoms often masked by peri-procedural opioids and, in retroperitoneal hematomas, by continued fluid sequestration within the hematoma [4].

Diagnosis is primarily clinical, based on the characteristic anterior-thigh distribution and sensory abnormalities, together with exclusion of hematoma or infection; ultrasound or MRI can serve as an adjunct to assess structural compression. Preventive measures include avoiding large-bore needles, minimizing catheter manipulation, and using ultrasound guidance [5]. Pivec et al. showed that ultrasound accurately identifies the IFCN and MFCN and localizes suspected neuropathy, with IFCN damage typically in the proximal thigh below the inguinal ligament and MFCN damage in the medial knee region, supporting a preventive role for ultrasound [6].

Management centers on symptom relief and nerve recovery. Repair of a femoral pseudoaneurysm presenting as a pulsatile mass can rapidly resolve symptoms, whereas hematoma drainage yields less consistent results, likely owing to diagnostic delay and extensive nerve damage [4]. Conservative care comprises analgesics, physiotherapy, and neurorehabilitation. Nerve hydro-dissection separates the entrapped nerve from surrounding fascial and soft tissues at areas of entrapment [7]; ultrasound guidance improves precision, efficacy, and safety compared with landmark-based approaches [8]. In nerve entrapment, compression of the epineural vasa vasorum disrupts the blood–nerve barrier, causing segmental intraneural ischemia and edema and ectopic impulse generation, and eventually demyelination and axonal loss; hydro-dissection relieves pressure on the nerve and on the neurovascular and lymphatic structures supplying it [7]. Most hydro-dissection evidence derives from carpal tunnel syndrome, the commonest entrapment [9]. Injectates, including saline, local anesthetics, steroids, 5% dextrose, platelet-rich plasma, and hyaluronidase, provide both mechanical release and anti-inflammatory effects, but no consensus exists on agent, volume, or frequency, and comparative evidence remains inconclusive because of small samples and heterogeneous protocols [9]. Surgical neurectomy may be considered for refractory cases but risks neuroma formation and recurrent or worsening symptoms [10]. Because the local anaesthetic within the perineural injection provides immediate diagnostic confirmation, we performed a combined diagnostic–therapeutic hydro-dissection in a single session rather than a separate diagnostic block, given the chronicity of symptoms, clear clinical localization, and prior failed pharmacotherapy. This report is limited by its small size and uncontrolled design, and a causal relationship between the intervention and outcome cannot be definitively established from two cases; nonetheless, the immediate response to local anaesthetic, the durable resolution beyond the anaesthetic duration, and the prior failure of pharmacotherapy support a treatment effect and warrant confirmation in larger, prospective studies.

## 6 Conclusion

AFCN neuralgia following femoral artery catheterization is an uncommon but clinically significant cause of persistent neuropathic pain and reduced quality of life. Awareness of the relevant anatomy, mechanisms, risk factors, and clinical presentation enables prompt recognition, conservative management, and prevention. Ultrasound-guided AFCN hydro-dissection provides targeted nerve decompression and facilitates neural recovery, achieving durable relief in both patients. Further research is warranted to optimize injectate selection and technique.

## References

[1] Damluji A.A., Nelson D.W., Valgimigli M. (2017). Transfemoral approach for coronary angiography and intervention: a collaboration of international cardiovascular societies. JACC Cardiovasc Interv.

[2] El-Ghanem M., Malik A.A., Azzam A., Yacoub H.A., Qureshi A.I., Souayah N. (2017). Occurrence of femoral nerve injury among patients undergoing transfemoral percutaneous catheterization procedures in the United States. J Vasc Interv Neurol.

[3] Bjørn S., Nielsen T.D., Jensen A.E. (2023). The anterior branch of the medial femoral cutaneous nerve innervates the anterior knee: a randomized volunteer trial. Minerva Anestesiol.

[4] Kent K.C., Moscucci M., Gallagher S.G., DiMattia S.T., Skillman J.J. (1994). Neuropathy after cardiac catheterization: incidence, clinical patterns, and long-term outcome. J Vasc Surg.

[5] Kuo F., Park J., Chow K., Chen A., Walsworth M.K. (2019). Avoiding peripheral nerve injury in arterial interventions. Diagn Interv Radiol.

[6] Pivec C., Bodner G., Mayer J.A. (2018). Novel demonstration of the anterior femoral cutaneous nerves using ultrasound. Ultraschall Med.

[7] Lam K.H.S., Hung C.Y., Chiang Y.P. (2020). Ultrasound-guided nerve hydrodissection for pain management: rationale, methods, current literature, and theoretical mechanisms. J Pain Res.

[8] Chang K.V., Wu W.T., Özçakar L. (2020). Ultrasound imaging and guidance in peripheral nerve entrapment: hydrodissection highlighted. Pain Manag.

[9] Buntragulpoontawee M., Chang K.V., Vitoonpong T. (2021). The effectiveness and safety of commonly used injectates for ultrasound-guided hydrodissection treatment of peripheral nerve entrapment syndromes: a systematic review. Front Pharmacol.

[10] Dalili D., Ahlawat S., Rashidi A., Belzberg A.J., Fritz J. (2021). Cryoanalgesia of the anterior femoral cutaneous nerve (AFCN) for the treatment of neuropathy-mediated anterior thigh pain: anatomy and technical description. Skelet Radiol.

